# Ongoing donor-transmitted diabetic kidney disease in kidney transplant recipients with fair sugar control: a single center retrospective study

**DOI:** 10.1186/s12882-020-02132-w

**Published:** 2020-11-03

**Authors:** Chia-Tien Hsu, Mei-Chin Wen, Hsien-Fu Chiu, Shang-Feng Tsai, Tung-Min Yu, Cheng-Kuang Yang, Ming-Ju Wu, Cheng-Hsu Chen

**Affiliations:** 1 Division of Nephrology, Department of Internal Medicine, Taichung Veterans General Hospital, Taichung, Taiwan; 2 Department of Pathology and Laboratory Medicine, Taichung Veterans General Hospital, Taichung, Taiwan; 3 Division of Urology, Department of Surgery, Taichung Veterans General Hospital, Taichung, Taiwan

**Keywords:** Donor-transmitted diabetic kidney disease, Kidney transplantation, Diabetes mellitus

## Abstract

**Background:**

Transplantation with a diabetic donor kidney may have some benefits compared to remaining on the waitlist for selected patients. However, we found that some kidney transplant recipients have ongoing donor-transmitted diabetic kidney disease (DT-DKD) despite fair blood sugar control. This study aimed to survey the incidence and clinical pattern of DT-DKD in kidney transplant recipients.

**Methods:**

We retrospectively reviewed the medical records of kidney transplantations in our hospital. We found 357 kidney transplantations from February 2006 to April 2018. Among these, 23 (6.4%) diabetic donor kidney transplantations were done in the study period.

**Results:**

Among the 23 recipients, 6 (26.1%) displayed biopsy-proven DKD. Recipients with biopsy-proven DKD had longer dialysis vintage, higher proteinuria amount, lower last estimated glomerular filtration rate (eGFR), and a more rapid decline in the eGFR. The median fasting blood sugar level in the biopsy-proven DKD group was unexpectedly lower than the non-DKD group. Most of the pre-implantation frozen sections in biopsy-proven DKD group showed diabetic lesions worse than diabetic nephropathy (DN) class IIa. In the biopsy-proven DKD group, 5 recipients had no history of diabetes before or after transplantation. Among the 23 recipients, 5 (21.7%) were diagnosed with DT-DKD. Serial post-transplant biopsies showed the histological progression of allograft DN.

**Conclusions:**

To the best of our knowledge, this is the first study to report the phenomenon of ongoing DT-DKD in kidney transplant recipients with fair blood sugar control. The zero-time pre-transplant kidney biopsy may be an important examination before the allocation of diabetic donor kidneys. Further study is needed to elucidate the possible mechanism of ongoing DT-DKD in non-diabetic recipients with fair blood sugar control as well as the impaction of pre-implantation diabetic lesion on the graft outcome.

**Supplementary Information:**

The online version contains supplementary material available at 10.1186/s12882-020-02132-w.

## Background

According a report by the United States Renal Data System, the incidence and prevalence of end-stage renal disease (ESRD) in Taiwan has persistently been one of the highest worldwide [1]; this has led to serious medical and socioeconomic problems in the country [2]. According to the statistical data of the Taiwan Organ Registry and Sharing Center, more than 7400 ESRD patients were waitlisted for kidney transplantation in 2018. However, only 352 kidney transplantations (including 181 deceased donor and 171 living donor kidney transplantations) were performed in 2018 [3]. The appropriate utilization of marginal or extended criteria donors is one of the possible solutions to the organ shortage crisis [4, 5]. Few studies have surveyed the impact of diabetes on the allocation of diabetic donor kidneys against the backdrop of the organ shortage situation. Compared to remaining on the waitlist or receiving a kidney from a non-diabetic extended criteria donor, transplantation with a diabetic donor kidney may have benefits for selected patients [6–9]. Some studies have shown the improvement of early diabetic lesion in the transplanted kidney if the recipient has good post-transplant glycemic control [10, 11]. However, we found that some kidney transplant recipients have biopsy-proven ongoing diabetic kidney disease (DKD) despite clinical euglycemia and no history of diabetes. We defined this phenomenon as donor-transmitted DKD (DT-DKD). The aim of this study was to survey the incidence and clinical pattern of DT-DKD in kidney transplant recipients.

## Methods

### Study design and subjects

**Fig. 1:**
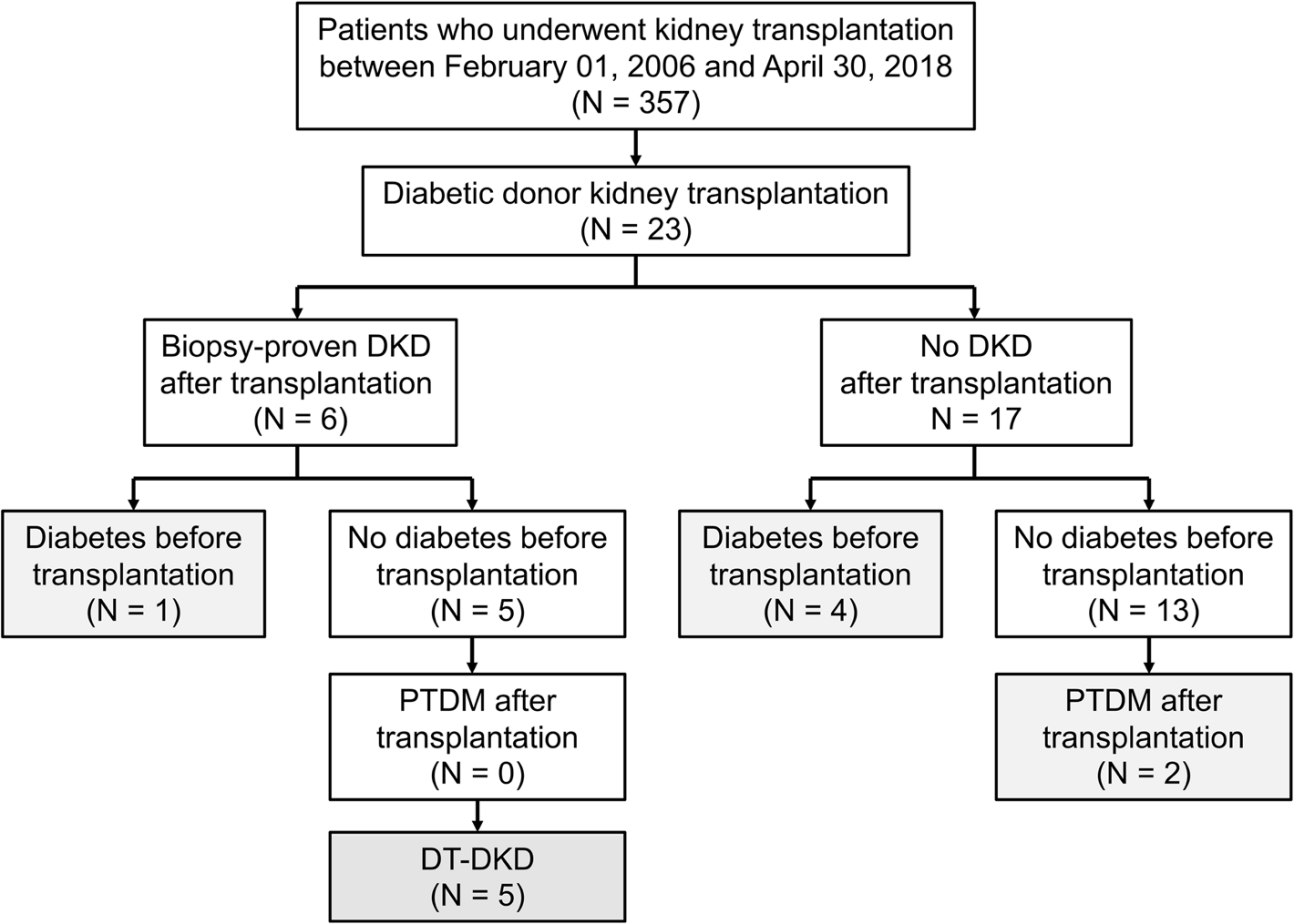
Flow diagram of the study patients. DT-DKD, donor-transmitted diabetic kidney disease; PTDM, post-transplant diabetes mellitus

We conducted a retrospective cohort study and reviewed the medical record of kidney transplantations at our centre between February 2006 and April 2018. A total of 357 kidney transplantations were performed in the study period. Among these, 23 (6.4%) diabetic donor kidney transplant recipients were included for data analysis (Fig. 1). Our study was approved by the institutional review board of Taichung Veterans General Hospital (IRB TCVGH No: CE20012B). Patient informed consent was waived due to the retrospective data analysis nature of this study.

Indication biopsies were done when there was delayed graft function, presence of donor-specific antibody, or an unexplained increase in serum creatinine or proteinuria. We reviewed medical records to obtain information on sex, age, medical history, anthropometric data, dialysis vintage, transplant medications, laboratory results, and pathologic reports. During the study period, 21 of the 23 diabetic donor kidney transplant recipients were found to have undergone at least one post-transplant allograft biopsy. A total of 61 follow-up biopsies (2.9 biopsies per patient) were examined. All of the allograft biopsies were examined using light microscopy, immunofluorescence studies, and electron microscopy. Silver methenamine, periodic acid-Schiff (PAS), and Masson trichrome stains were done for light microscopic examinations. IgG, IgA, IgM, C3, C4d, C1q, kappa light chains, lambda light chains, and simian virus 40 immunohistochemical stains were done for immunofluorescence studies. The test of anti-HLA antibodies using the Luminex® method is expensive and is not affordable to every patient in our cohort. Nine recipients underwent the anti-HLA antibody test in our cohort. We summarized the findings of post-transplant indication biopsies in the supplementary Table 1.

Based on the clinical data and histopathologic results, the 23 diabetic donor kidney transplant recipients were divided into two groups: the biopsy-proven DKD group (*n* = 6) and non-DKD group (no biopsy-proven DKD or no clinical suspicion of DKD; *n* = 17). One recipient in the biopsy-proven DKD group had a history of diabetes before transplantation, and 4 in the non-DKD group had history of diabetes before transplantation. In the non-DKD group, 2 recipients were found to have post-transplant diabetes mellitus (PTDM). All the results of glycated hemoglobin (HbA1c) beyond 3 months post-transplantation were included to calculate the HbA1c after transplantation. Besides, the fasting blood sugar levels after transplantation of all 23 recipients were included to draw the box and whisker plot for evaluating the blood sugar level distribution. The estimated glomerular filtration rate (eGFR) was calculated via the Modification of Diet in Renal Disease Study equation [12]. Overweight is defined as body mass index (BMI) ≧ 24 kg/m^2^ by the Department of Health in Taiwan.

### Statistical analysis

Data are shown as the mean ± standard deviation or median (first quartile, third quartile) for continuous variables according to their distribution, and as number (percentage) for categorical variables. The assessment of normality was conducted using the Kolmogorov-Smirnov test and Shapiro-Wilk test. Statistical analyses were performed using MedCalc for Windows, version 15.0 (MedCalc Software, Ostend, Belgium). Tests for statistical significance were conducted using the Mann-Whitney U test for continuous variables, and the Fisher’s exact test or Chi-Squared Test for categorical variables. A *p* value < 0.05 was considered statistically significant.

## Results

There were 9 male and 14 female diabetic donor kidney transplant recipients with a median age of 44.2 years. The median follow-up duration was 4.4 years. The median baseline estimated glomerular filtration rate (eGFR) after kidney transplantation was 62.7 ml/min/1.73m^2^, while the last eGFR was 37.7 ml/min/1.73m^2^.

At last follow-up, 6 (26.1%) of the 23 recipients displayed biopsy-proven DKD in the transplanted kidneys. Based on the clinical data and histopathologic results, the selected recipients were divided into two groups: the biopsy-proven DKD group (*n* = 6) and non-DKD group (*n* = 17). Table 1 shows the clinical characteristics and comparison of the two groups. Compared with the non-DKD group, the recipients with biopsy-proven DKD group had a longer dialysis vintage (5.9 vs. 2.8 years; *p* = 0.005), higher proteinuria amount (7.3 vs. 0.2 mg/mg; *p* = 0.001), lower last eGFR (10.4 vs. 47.3 ml/min/1.73m^2^; *p* = 0.003), and a more rapid decline in the eGFR (10.6 vs. 5.8 ml/min/1.73m^2^/year; *p* = 0.017). In the biopsy-proven DKD group, more recipients had dyslipidemia at the time of follow-up indication biopsy (100% vs. 41.2%; *p* = 0.019). One graft failure due to DKD was noted in the biopsy-proven DKD group.

**Table 1:** Clinical characteristics of the 23 diabetic donor kidney transplant recipients

	**Recipients with biopsy-proven DKD** **(*n*** **= 6)**		**Recipients** **without DKD** **(*n*** **= 17)**		***p*** **value**
*Recipient characteristics*					
Male sex†	4	(66.7%)	5	(29.4%)	0.162
Median age at transplant in years‡	42.5	(32.8–44.2)	46.7	(36.5–52)	0.327
Pre-transplant dialysis†	6	(100%)	13	(76.5%)	0.539
Median years on dialysis‡	5.9	(3.7–9.2)	2.8	(1.8–4.5)	0.005**
Cause of ESRD§					0.854
Diabetes†	1	(16.7%)	4	(23.5%)	1.000
Glomerular disease†	2	(33.3%)	7	(41.2%)	1.000
Other†	1	(16.7%)	1	(5.9%)	0.462
Unknown†	2	(33.3%)	5	(29.4%)	1.000
Median BMI (kg/m^2^) at transplant‡	23.1	(21.1–24.9)	22.3	(20.8–25.2)	0.806
Anti-HCV (+) recipient†	0	(0%)	2	(11.8%)	1.000
Post-transplant diabetes mellitus†	0	(0%)	2	(11.8%)	1.000
HbA1c before transplantation (%) ‡	5.4	(5.2–5.5)	5.4	(5.0–6.1)	0.889
HbA1c after transplantation (%) ‡	5.9	(5.7–5.9)	6.0	(5.5–6.9)	0.674
Median BMI (kg/m^2^) at follow-up biopsy‡	24.8	(21.6–26.7)	21.7	(20.0–25.2)	0.183
Weight change (kg) after transplantation ‡	−1.5	(−2.7–4.6)	−1.0	(−3.1–1.8)	0.861
Overweight at follow-up biopsy†	3	(50.0%)	5	(29.4%)	0.621
HbA1c≧5.7% at follow-up biopsy †	5	(83.3%)	12	(70.6%)	1.000
Dyslipidemia at follow-up biopsy†	6	(100%)	7	(41.2%)	0.019*
Hypertension at follow-up biopsy†	6	(100%)	12	(70.6%)	0.273
Last UPCR after transplantation (mg/mg) ‡	7.3	(2.0–10.0)	0.2	(0.1–0.7)	0.001**
Best eGFR after transplantation (ml/min/1.73m^2^) ‡	49.0	(42.2–85.1)	62.9	(53.3–81.2)	0.294
Last eGFR after transplantation (ml/min/1.73m^2^) ‡	10.4	(8.9–14.2)	47.3	(32.3–55.2)	0.003**
eGFR decline rate (ml/min/1.73m^2^/year) ‡	10.6	(8.7–14.9)	5.8	(2.6–9.8)	0.017*
Median duration of follow-up in years‡	4.2	(3.7–7.3)	5.6	(2.7–10.5)	0.575
Graft failure†	1	(16.7%)	0	(0%)	0.261
*Donor characteristics*					
Median donor age in years ‡	50.5	(39.0–57.0)	54.0	(37.5–60.0)	0.529
Median BMI at transplant (kg/m^2^) ‡	26.9	(24–28.2)	23.9	(22.6–30.8)	0.506
Creatinine at transplant (mg/dl) ‡	1.7	(0.8–2.5)	1.0	(0.7–1.4)	0.345
eGFR at transplant (ml/min/1.73m^2^) ‡	60.1	(28.4–111.6)	84.1	(56.3–99.8)	0.649
Last HbA1c before transplantation (%) ‡	7.1	(7.0–8.1)	6.7	(6.3–7.1)	0.069
Anti-HCV (+) donor†	0	(0%)	2	(11.8%)	1.000
History of hypertension†	4	(66.7%)	6	(35.3%)	0.341
Expanded criteria donor†	3	(50%)	7	(41.2%)	1.000
Transplanted kidney RPS DN classification≧IIa †	4/4^**a**^	(100%)	0/7^**b**^	(14.3%)	0.003*
*Transplant characteristics*					
PRA≧30%†	1	(16.7%)	6	(35.3%)	0.621
≧1 HLA mismatch†	5	(83.3%)	16	(94.1%)	0.462
Delayed graft function†	3	(50.0%)	3	(17.6%)	0.279
Acute rejection within 1 year†	2	(33.3%)	4	(23.5%)	0.632
Tacrolimus + MMF/MPA + steroid maintenance†	5	(83.3%)	13	(76.5%)	1.000
Cyclosporine + MMF/MPA + steroid maintenance†	1	(16.7%)	4	(23.5%)	1.000
No induction†	1	(16.7%)	1	(5.9%)	0.462
Lymphodepleting induction†	2	(33.3%)	6	(35.3%)	1.000
Non-lymphodepleting induction†	3	(50.0%)	10	(58.8%)	1.000

^**a**^ Four kidney transplantations have pre-implantation frozen sections in the biopsy-proven DKD group

^**b**^ Seven kidney transplantations have pre-implantation frozen sections in the non-DKD group

*p < 0.05; **p < 0.01. †Fisher’s exact test. ‡Mann–Whitney U-test. §Chi-Squared Test. Values are expressed as Number (percentage) or Median (Interquartile range). *DT-DKD* Donor-transmitted diabetic kidney disease; *ESRD* End-stage renal disease; *BMI* Body mass index; *HCV* Hepatitis C virus; *HbA1c* Glycated hemoglobin; *UPCR* Urine protein-to-creatinine ratio; *eGFR* Estimated glomerular filtration rate; *RPS DN class* Renal Pathology Society classification of diabetic nephropathy; *PRA* Panel-reactive antibody; *HLA* Human leukocyte antigen; *MMF* Mycophenolate mofetil; *MPA* Mycophenolic acid

Zero-time pre-transplant kidney biopsies were done in 11(47.8%) of the 23 kidney transplantations. Pre-implantation frozen sections were available in 4 and 7 cases in the biopsy-proven DKD group and the non-DKD group, respectively. All the 4 frozen sections in the biopsy-proven DKD group showed diabetic lesions worse than diabetic nephropathy (DN) class IIa lesion (class IIa, IIb, IIb, IIb, respectively), as defined by the Renal Pathology Society [13]. However, none of the 7 frozen sections in the non-DKD group displayed DN lesion worse than DN class IIa (*p* = 0.003). Other findings of zero-time pre-transplant kidney biopsies included moderate interstitial fibrosis and tubular atrophy (IFTA) 30% (1 frozen section in the non-DKD group), mild IFTA < 25% (3 frozen sections in the non-DKD group and 4 frozen sections in the biopsy-proven DKD group), mild arterionephrosclerosis (4 frozen sections in the non-DKD group and 4 frozen sections in the biopsy-proven DKD group), and acute tubular necrosis (1 frozen section in the biopsy-proven DKD group).

**Fig. 2:**
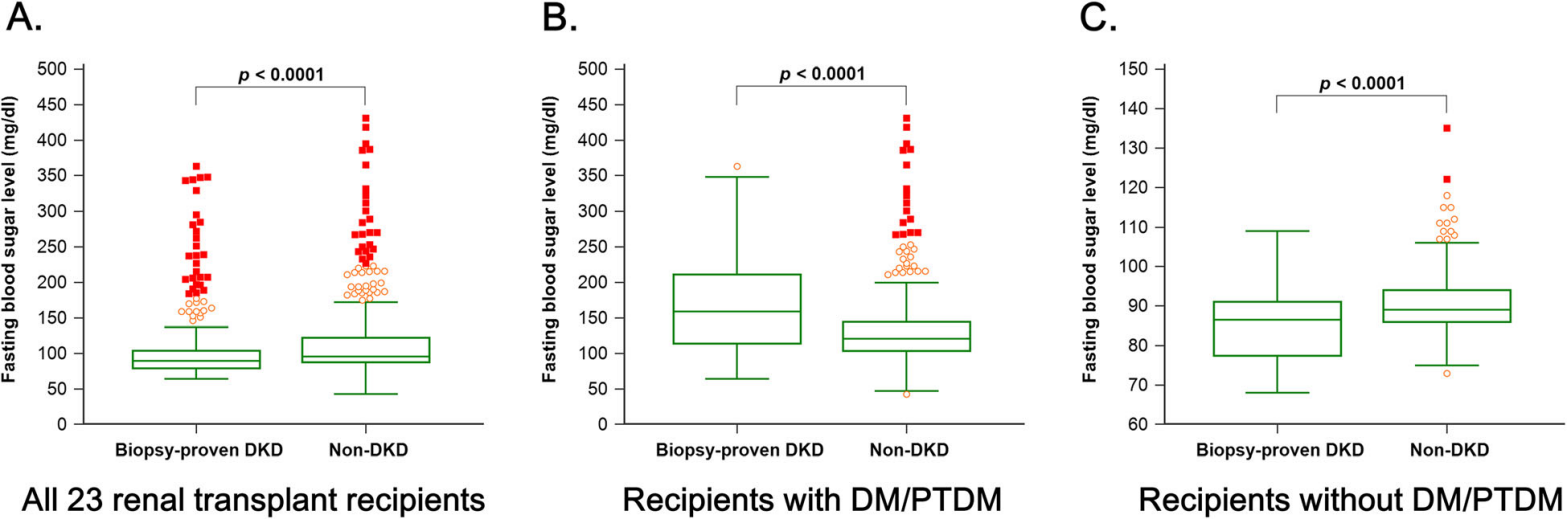
**a**. The fasting blood sugar level distribution of the 23 diabetic donor kidney transplant recipients. **b**. The fasting blood sugar level distribution of the recipients with diabetes. **c**. The fasting blood sugar level distribution of the recipients without diabetes. PTDM, post-transplant diabetes mellitus

Fig. 2 presents the fasting blood sugar level distribution of the 23 diabetic donor kidney transplant recipients. The median fasting blood sugar level in the biopsy-proven DKD group was unexpectedly lower than those in the non-DKD group (90 vs. 96 mg/dl; *p* < 0.0001; Fig. 2a). Subgroup analyses show that the median fasting blood sugar level in the biopsy-proven DKD group was higher among the recipients with diabetes (159 vs. 121 mg/dl; *p* < 0.0001; Fig. 2b), and lower among the recipients without diabetes (86.5 vs. 89 mg/dl; *p* < 0.0001; Fig. 2c).

In the biopsy-proven DKD group, 5 recipients had no history of diabetes before or after transplantation. Hence, 5 (21.7%) of the 23 recipients were diagnosed with DT-DKD. Table 2 shows the clinical characteristics of the 5 recipients with DT-DKD. Serial post-transplant biopsies showed the progression of allograft diabetic lesion.

**Table 2:** Clinical characteristics of 5 recipients with ongoing donor-transmitted diabetic kidney disease

**Case number**	1	2	3	4	5
*Recipient characteristics*					
Sex	Female	Male	Male	Male	Female
VAge in years	32.8	44.2	52.7	27.5	43.6
Pre-transplant dialysis	HD	HD	HD	HD	HD
Years on dialysis	11.4	6.8	3.7	3.7	5.0
Cause of ESRD	GN	Unknown	Analgesic	SLE	Unknown
BMI at transplant (kg/m^2^)	27.5	24.9	24.6	20.3	21.1
Anti-HCV (+) recipient	N	N	N	N	N
Post-transplant diabetes mellitus	N	N	N	N	N
BMI at follow-up biopsy (kg/m^2^)	25.9	26.7	23.7	28.0	20.0
Weight change (kg) after transplantation	−4.5	+ 4.6	−2.3	+ 24.9	−2.7
Dyslipidemia at follow-up biopsy	Y	Y	Y	Y	Y
Hypertension at follow-up biopsy	Y	Y	Y	Y	Y
HbA1c before transplantation (%)	5.3	5.2	5.5	5.2	5.4
HbA1c after transplantation (%)	5.3	5.8	5.7	5.9	5.9
Last UPCR after transplantation (mg/mg)	11.626	5.379	1.980	0.849	10.03
Best eGFR after transplantation (ml/min/1.73m^2^)	40.33	57.79	42.16	143.78	44.30
Last eGFR after transplantation (ml/min/1.73m^2^)	14.16	11.51	9.28	39.71	5.4
eGFR decline rate (ml/min/1.73m^2^/year)	8.72	6.77	10.86	14.87	10.30
Duration of follow-up in years	3.7	7.4	3.2	7.5	4.0
Allograft biopsy RPS DN classification	IIa → IIb → III^a^	III	III	IIb → III^a^	IIb → III → IV^a^
Number of indication follow-up biopsies	5	4	2	4	3
Timing of each biopsy/RPS DN classification					
(post-transplant months/DN classification)	0.2/IIa	10.7/ III	18.5/ III	20.6/IIb	10.2/IIb
(post-transplant months/DN classification)	0.6/IIa	21.2/ III	36.0/ III	62.9/III	20.1/III
(post-transplant months/DN classification)	3.5/IIa	61.7/ III		79.5/III	34.8/IV
(post-transplant months/DN classification)	13.9/IIb	65.5/ III		83.2/III	
(post-transplant months/DN classification)	26.4/III				
Graft failure	N	N	N	N	Y
*Donor characteristics*					
Donor age in years	39	57	57	22	44
Body mass index at transplant (kg/m^2^)	26.0	28.2	28.2	27.7	22.9
Creatinine at transplant (mg/dl)	2.8	2.5	2.5	0.5	0.8
eGFR at transplant (ml/min/1.73m^2^)	26.95	28.44	28.44	163.98	111.62
Last HbA1c before transplantation (%)	8.0	7.0	7.0	7.1	6.3
Anti-HCV (+) donor	N	N	N	N	N
Expanded criteria donor	N	Y	Y	N	N
Zero-time biopsy RPS DN classification	IIa	IIb	IIb	Nil	Nil
*Transplant characteristics*					
PRA≧30%	N	N	N	N	Y
≧1 HLA mismatch	Y	Y	Y	N	Y
Delayed graft function	Y	N	N	N	Y
Acute rejection within 1 year	Y	N	N	N	Y
Tacrolimus + MMF/MPA + steroid maintenance	Y	Y	Y	Y	Y
No induction	N	N	N	Y	N
Lymphodepleting induction	Y	N	N	N	N
Non-lymphodepleting induction	N	Y	Y	N	Y

^a^Serial post-transplant biopsies showed the progression of allograft diabetic lesion. *Y* Yes; *N* No; *Nil* Not performed; *HD* Hemodialysis, *ESRD* End-stage renal disease; *GN* Glomerulonephritis; *SLE* Systemic lupus erythematosus; *BMI* Body mass index; *HCV* Hepatitis C virus; *HbA1c* Glycated hemoglobin; *UPCR* Urine protein-to-creatinine ratio; *eGFR* Estimated glomerular filtration rate; *RPS DN classification* Renal Pathology Society classification of diabetic nephropathy; *PRA* Panel-reactive antibody; *HLA* Human leukocyte antigen; *MMF* Mycophenolate mofetil; *MPA* Mycophenolic acid

## Discussion

To the best of our knowledge, this is the first study to report the phenomenon of ongoing DT-DKD in non-diabetic kidney transplant recipients with fair blood sugar control. Few studies have previously evaluated the reversal of DN after transplantation into non-diabetic recipients. Abouna et al. [10] reported a deceased donor with a 17-year history of type 1 diabetes. Pre-transplant histological examination showed features of DN including thickening of basement membranes and diffuse glomerulosclerosis. After transplantation into two non-diabetic recipients, reversal of DN was found at 7 months post-transplantation in both transplant kidneys. Harada et al. [11] also reported the reversal of early stage DN in grafts from three living diabetic donors after transplantation into non-diabetic recipients. In our study, 11 of 23 kidney transplantations had undergone pre-transplant histological examinations. 7 pre-implantation frozen sections showed no obvious DN. However, we cannot rule out the possible RPS class I DN in these transplant kidneys because some class I DN kidneys showed glomerular basement membranes thickening only when examined using electron microscopy. Therefore, we cannot exclude the possible reversal of early stage DN in some recipients of our cohort.

Recently, Truong et al. [14] and Khan et al. [15] reported 26 diabetic donor kidney transplantations with post-perfusion biopsies and follow-up biopsies. All the donors in their study with dipstick proteinuria < 2+ (< 300 mg/g) showed diabetic lesions equal or lower than RPS IIa category on pre-implantation biopsy. Among their study subjects, 2 transplanted kidneys with class IIa DN progressed slowly (class IIa to IIb), but this progress was related to recipient diabetes in both cases. They also reported that one transplanted kidney without DN before transplantation developed class IIa DN in a recipient with PTDM. In their cohort, most transplanted kidneys did not show progression of diabetic lesions. In our study, 4 pre-implantation frozen sections in the biopsy-proven DKD group showed diabetic lesions worse than DN class IIa. Besides, all post-transplant allograft biopsies of the 6 recipients in our biopsy-proven DKD group showed progression of DN. Our study showed that some transplanted kidneys with later stages of DN may worsen after transplantation into non-diabetic recipients, even if the recipients are non-diabetic and with fair post-transplant blood sugar control. Okada et al. [16] reported early graft loss (16 months and 20 months after transplantation) in two non-diabetic recipients of mate-kidneys from the same diabetic donor. Pre-transplant biopsy in their study showed DN with nodular sclerosis (class III DN). Hence, zero-time biopsy may be an important examination to precisely evaluate the status of DN before allocation of diabetic donor kidneys.

The urine dipstick proteinuria or urinary protein creatinine ratio (UPCR) test may be a good screening test before pre-implantation biopsy. Truong et al. [14] and Khan et al. [15] reported that all their study donors with dipstick proteinuria < 2+ (< 300 mg/g) showed diabetic lesions equal or lower than RPS IIa category on pre-implantation biopsy. In our study, two donors with 2+ dipstick proteinuria were transplanted into three recipients in the biopsy proven DKD group. All of them showed RPS IIb diabetic lesions in the pre-implantation biopsies. However, many deceased donors did not have at least two urine dipstick or UPCR measurements separated by at least 3 months. We could not rule out transient proteinuria caused by acute hemodynamic change, such as hypovolemia or epinephrine administration. In the non-DKD group of our cohort, there are two donors with initially proteinuria ≧2+, but the follow-up urine dipstick tests showed proteinuria < 2+ before organ procurement. Serial urine dipstick measurements or further biopsy may be needed to determine the allocation of kidneys.

In our study, the median fasting blood sugar level in the biopsy-proven DKD group was unexpectedly lower than in the non-DKD group. This may be related to the poorer renal function in the DKD group. Chronic kidney disease (CKD) is a risk factor for low blood sugar level in patients with or without diabetes [17]. There are many possible mechanisms to explain the association between CKD and low blood sugar level, such as decreased insulin renal clearance, impaired renal gluconeogenesis, diminished insulin degradation, and poor nutrition. Patients with CKD and diabetes are at risk for both hypoglycemia and hyperglycemia [18]. Subgroup analyses in our study showed that the median fasting blood sugar level in the biopsy-proven DKD group was higher among the recipients with diabetes. Chronic inflammation-related insulin resistance as well as decreased glucose filtration and excretion are the possible reasons for hyperglycemic episodes.

Some limitations of this study should be acknowledged. We did not perform oral glucose tolerance test (OGTT) in these patients. Some patients could have had diabetes already but had normal fasting sugar or HbA1c. The retrospective nature of the study may have led to some unrecognized confounding factors to bias the findings. Besides, the case number in this study is small. Nevertheless, this study represents real-world conditions. Diabetic donor kidneys account for 6.4% of kidney transplantations in our hospital. This frequency is similar to that reported in studies (3.5, 6.4, 5.6, and 6.1%) by Ahmad, Mohan, Cohen, and Truong, respectively [7–9, 14]. One graft failure, higher proteinuria amount, a more rapid decline in the eGFR, and ongoing DKD were noted in the biopsy-proven DKD group in our study. Previous studies showed that diabetic donor kidneys with early stage of DN (equal or lower than class IIa) may have little adverse effect on graft survival, and DN may progress slowly or may stabilize [14, 15]. Our findings differ from those of this study, as DN progress with poorer renal outcomes noted in recipients with DT-DKD. The diabetic lesions could appear and progress in the presence of diabetes, extreme obesity, prediabetes, and other elements of the metabolic syndrome [19]. Our survey also showed that the progression of graft diabetic lesions may be related to the severity of donor DN lesion. Diabetic donors with diabetic lesions worse than diabetic DN class IIb may be not suitable for renal transplantation. Even in patients with an adequate glucose control, the histological lesions progressed. This hypothesis will need further studies with larger sample size.

## Conclusions

To the best of our knowledge, this is the first study to report the phenomenon of ongoing DT-DKD in kidney transplant recipients with fair blood sugar control. The progression of graft diabetic lesions may be related to the severity of donor DN lesion. The zero-time pre-transplant kidney biopsy may be an important examination before the allocation of diabetic donor kidneys. Further study is needed to elucidate the possible mechanism of ongoing DT-DKD in non-diabetic recipients with fair blood sugar control, and the impaction of pre-implantation diabetic lesion on the graft outcome.

## Supplementary Information

**Additional file 1.**

## Data Availability

The individual patient-level data was not made publicly available due to containing potentially identifying patient data; however, the study data may be made available from the authors upon reasonable request.

## Abbreviations

DT-DKD: Donor-transmitted diabetic kidney disease;
eGFR: Estimated glomerular filtration rate
DN: Diabetic nephropathy
ESRD: End-stage renal disease
PTDM: Post-transplant diabetes mellitus
HbA1c: Glycated hemoglobin
CKD: Chronic kidney disease

## References

[1] United States Renal Data System. 2019 USRDS annual data report. Epidemiology of kidney disease in the United States. National Institutes of Health, National Institute of Diabetes and Digestive and Kidney Diseases, Bethesda, MD, 2019.

[2] Yang WC, Hwang SJ (2008). Taiwan Society of Nephrology. Incidence, prevalence and mortality trends of dialysis end-stage renal disease in Taiwan from 1990 to 2001: the impact of national health insurance. Nephrol Dial Transplant.

[3] Taiwan Organ Registry and Sharing Center. https://www.torsc.org.tw/FileUploads/docatt/97790404-b4e7-4b8b-95f4-39aeb3c207ec.pdf, and https://www.torsc.org.tw/FileUploads/docatt/4836561f-0fde-0356-2f1c-fa37da3768f3.pdf. Accessed 01 Nov 2020.

[4] Abouna GM (2008). Organ shortage crisis: problems and possible solutions. Transplant Proc.

[5] Heilman RL, Mathur A, Smith ML, Kaplan B, Reddy KS (2016). Increasing the use of kidneys from unconventional and high-risk deceased donors. Am J Transplant.

[6] Cohen JB, Eddinger KC, Locke JE, Forde KA, Reese PP, Sawinski DL (2017). Survival benefit of transplantation with a deceased diabetic donor kidney compared with remaining on the waitlist. Clin J Am Soc Nephrol.

[7] Cohen JB, Bloom RD, Reese PP, Porrett PM, Forde KA, Sawinski DL (2016). National outcomes of kidney transplantation from deceased diabetic donors. Kidney Int.

[8] Mohan S, Tanriover B, Ali N, Crew RJ, Dube GK, Radhakrishnan J (2012). Availability, utilization and outcomes of deceased diabetic donor kidneys; analysis based on the UNOS registry. Am J Transplant.

[9] Ahmad M, Cole EH, Cardella CJ, Cattran DC, Schiff J, Tinckam KJ (2009). Impact of deceased donor diabetes mellitus on kidney transplant outcomes: a propensity score-matched study. Transplantation.

[10] Abouna GM, Al-Adnani MS, Kremer GD, Kumar SA, Daddah SK, Kusma G (1983). Reversal of diabetic nephropathy in human cadaveric kidneys after transplantation into non-diabetic recipients. Lancet.

[11] Harada S, Ushigome H, Nishimura A, Nakao T, Nakamura T, Koshino K (2015). Histological reversibility of diabetic nephropathy after kidney transplantation from diabetic donor to non-diabetic recipient. Nephrology (Carlton).

[12] Poggio ED, Wang X, Weinstein DM, Issa N, Dennis VW, Braun WE (2006). Assessing glomerular filtration rate by estimation equations in kidney transplant recipients. Am J Transplant.

[13] Tervaert TW, Mooyaart AL, Amann K, Cohen AH, HT C, Drachenberg CB (2010). Renal Pathology Society. Pathologic classification of diabetic nephropathy. J Am Soc Nephrol.

[14] Truong LD, Suki WN, Gaber LW, Gaber OA, Khan F (2019). Kidney donors with diabetes: renal biopsy findings at time of transplantation and their significance. Transplant Direct.

[15] Khan FN, Truong LD, Nguyen DT, Gravoss EA, Bhatti MI, Frost AE (2020). Outcomes of kidney transplantation using deceased donors with history of diabetes. Clin Transpl.

[16] Okada D, Okumi M, Tanabe K (2017). Early graft loss in two recipients of mate kidneys from the same donor with diabetic nephropathy. Ther Apher Dial.

[17] Moen MF, Zhan M, Hsu VD, Walker LD, Einhorn LM, Seliger SL (2009). Frequency of hypoglycemia and its significance in chronic kidney disease. Clin J Am Soc Nephrol.

[18] Pecoits-Filho R, Abensur H, Betônico CC, Machado AD, Parente EB, Queiroz M (2016). Interactions between kidney disease and diabetes: dangerous liaisons. Diabetol Metab Syndr.

[19] Maric C, Hall JE (2011). Obesity, metabolic syndrome and diabetic nephropathy. Contrib Nephrol.

