# Supplementary material for: Ongoing donor-transmitted diabetic kidney disease in kidney transplant recipients with fair sugar control: a single center retrospective study
Source: BMC Nephrol. 2020 Nov 3;21:458. doi: 10.1186/s12882-020-02132-w (PMC7640448; doi:10.1186/s12882-020-02132-w)
Supplement: Supplementary file 1 — Additional file 1. [file 12882_2020_2132_MOESM1_ESM.docx]

| **Supplementary table 1. Renal transplant biopsy findings of post-transplant indication biopsies** | | | | | | | | | |
| --- | --- | --- | --- | --- | --- | --- | --- | --- | --- |
| Case number | Group | Number of biopsies | History of  rejection episode | Other findings | DSA | Banff lesion scores of last biopsy | | | |
|  |  |  |  |  |  | Arteriolar hyalinosis | Vascular  intimal thickening | IFTA | C4d |
| 1 | DKD | 5 | Active AMR |  | Positive | 3 | 1 | 2 | 0 |
| 2 | DKD | 4 | No rejection |  | Nil | 3 | 1 | 2 | 0 |
| 3 | DKD | 2 | No rejection |  | Nil | 3 | 1 | 1 | 0 |
| 4 | DKD | 4 | Acute TCMR |  | Nil | 0 | 0 | 1 | 0 |
| 5 | DKD | 3 | Acute TCMR |  | Nil | 3 | 0 | 1 | 0 |
| 6 | DKD | 4 | Acute TCMR |  | Nil | 3 | 1 | 2 | 0 |
| 7 | Non-DKD | 2 | Active AMR |  | Positive | 1 | 0 | 0 | 0 |
| 8 | Non-DKD | 3 | Active AMR |  | Negative | 1 | 0 | 1 | 0 |
| 9 | Non-DKD | 3 | Acute TCMR |  | Negative | 3 | 1 | 1 | 0 |
| 10 | Non-DKD | 4 | Acute TCMR |  | Negative | 0 | 0 | 2 | 0 |
| 11 | Non-DKD | 3 | Chronic active AMR |  | Nil | 1 | 1 | 1 | 0 |
| 12 | Non-DKD | 1 | Acute TCMR |  | Nil | 0 | 1 | 1 | 0 |
| 13 | Non-DKD | 3 | No rejection |  | Nil | 3 | 1 | 1 | 0 |
| 14 | Non-DKD | 1 | Acute TCMR |  | Nil | 3 | 1 | 1 | 0 |
| 15 | Non-DKD | 1 | Acute TCMR |  | Nil | 0 | 0 | 0 | 0 |
| 16 | Non-DKD | 5 | Acute TCMR | BKN | Negative | 1 | 2 | 1 | 0 |
| 17 | Non-DKD | 3 | Chronic active AMR |  | Negative | 1 | 1 | 1 | 0 |
| 18 | Non-DKD | 3 | No rejection | r-IgAN | Negative | 1 | 0 | 1 | 0 |
| 19 | Non-DKD | 1 | Active AMR |  | Nil | 1 | 0 | 2 | 3 |
| 20 | Non-DKD | 0 |  |  | Nil |  |  |  |  |
| 21 | Non-DKD | 4 | Chronic active AMR | r-IgAN | Nil | 2 | 1 | 1 | 0 |
| 22 | Non-DKD | 0 |  |  | Negative |  |  |  |  |
| 23 | Non-DKD | 2 | No rejection | r-LN | Nil | 2 | 1 | 1 | 0 |
| DKD, diabetic kidney disease; AMR, antibody medicated rejection; TCMR, T-cell medicated rejection; GN, glomerulonephritis; BKN, BK-virus nephropathy; r-IgAN, recurrent IgA nephropathy; r-LN, recurrent lupus nephritis; DSA, donor specific antibodies; IFTA, interstitial fibrosis and tubular atrophy. | | | | | | | | | |
